# 4‐Octyl itaconate blocks GSDMB‐mediated pyroptosis and restricts inflammation by inactivating granzyme A

**DOI:** 10.1111/cpr.13711

**Published:** 2024-07-09

**Authors:** Wenbin Gong, Hangyu Fu, Kui Yang, Tao Zheng, Kun Guo, Wei Zhao

**Affiliations:** ^1^ Department of General Surgery The First Affiliated Hospital of Xi'an Jiaotong University Xi'an China; ^2^ Department of General Surgery, Shanghai General Hospital Shanghai Jiao Tong University School of Medicine Shanghai China; ^3^ Department of General Surgery, the First Affiliated Hospital of USTC, Division of Life Sciences and Medicine University of Science and Technology of China Hefei China

## Abstract

GSDMB‐mediated pyroptosis facilitates a pro‐inflammatory immune microenvironment and needs to be tightly regulated to avoid excessive inflammation. Here, we provide evidence that itaconate and its cell‐permeable derivative 4‐octyl itaconate (4‐OI) can significantly inhibit GSDMB‐rendered pyroptotic activity independent of Nrf2. 4‐OI interferes proteolytic process of GSDMB by directly modifying Cys54, Cys148 and Ser212 on granzyme A (GrzA), a serine protease that site‐specifically cleaves the inter‐domain linker of GSDMB, instead of interaction with GSDMB, thereby blocking pyroptosis and exerts anti‐inflammatory effects. Moreover, 4‐OI alleviates inflammation by suppressing GSDMB‐induced pyroptotic cell death during acute colitis models in intestinal epithelial GSDMB conditional transgenic mice. Our data expand the role of 4‐OI as a crucial immunometabolic derivative that regulates innate immunity and inflammation through a newly identified posttranslational modification, and targeting of pyroptosis by 4‐OI therefore holds potent therapeutic potential for primarily inflammatory and/or autoimmune diseases.

## Graphical abstract

4‐octyl itaconate interferes proteolytic process of GSDMB by directly modifying Cys54, Cys148 and Ser212 on granzyme A, instead of interaction with GSDMB, thereby blocking pyroptosis and exerts anti‐inflammatory effects.

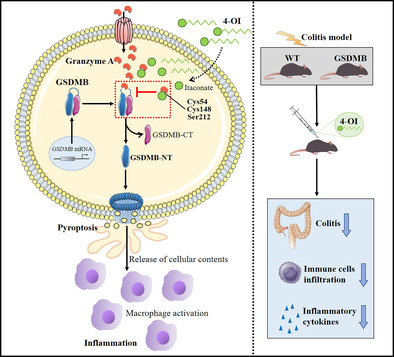

## 1 INTRODUCTION

Pyroptosis, an inflammatory form of programmed cell death that releases substantial cellular contents to activate fierce inflammation, is vital for immune defence against endogenous dangers and pathogenic infections.^1^ Pyroptosis is mediated by gasdermin (GSDM) family proteins that consist of an N‐terminal pore‐forming domain (GSDM‐NT), a C‐terminal autoinhibitory domain (GSDM‐CT) and an interdomain linker.^2^ The GSDMs, except for DFNB59 (PJVK), share a similar activation mechanism that proteolytic cleavage by specific proteases liberate GSDM‐NT fragment from autoinhibition to form oligomeric pores in membrane and execute subsequent lytic cell death.^3^ GSDMB is a distinctive member among the GSDM family as it is absent in rodents, and is cleaved by granzyme A (GrzA) derived from cytotoxic lymphocytes at Lys244 site in the interdomain linker to induce pyroptosis.^4^ GSDMB has six splicing isoforms and GSDMB isoforms 3, 4 and 6 containing exon 6 have been demonstrated to trigger normal pyroptotic activity whereas other isoforms 1, 2 and 5 with a deleted or modified exon 6 do not.^5^, ^6^, ^7^, ^8^ Intriguingly, GSDMB isoforms 1–3 have been found to be the most abundant isoforms among several tumour cell lines and isoforms 3 and 4 are often up‐regulated in tumours.^7^ Furthermore, GSDMB isoform 3 is shown to be the principal isoform in intestinal mucosal epithelial cells.^5^ Given that GSDMB isoform 3 has the strongest ability to elicit pyroptotic permeabilising activity,^5^, ^7^, ^8^ indicating isoform 3 may play an important role for immune system response to stressors. Of note, an excessive inflammatory response caused by pyroptosis can damage the host organism and aggravate the inflammation‐associated diseases, such as asthma, inflammatory bowel disease and even lethal septic shock.^2^ The inhibition of pyroptosis may therefore be the most efficacious strategy for treating such diseases that are primarily inflammatory or autoimmune.

Numerous studies have demonstrated that inflammatory response alters cell metabolism and in turn, cell metabolism also affects inflammatory response. The Krebs cycle‐derived metabolite itaconate has recently emerged as an important regulator of immunity and inflammation, with studies underlining its immunomodulatory effects in diverse contexts.^9^ Itaconate is synthesised from the decarboxylation of the Krebs cycle intermediate cis‐aconitate by the enzyme encoded by immune‐responsive gene 1 (IRG1), also termed ACOD1.^10^ Endogenous itaconate and/or its cell‐permeable derivative 4‐octyl itaconate (4‐OI) have been verified to exert the anti‐inflammatory properties via alkylation of cysteine residues on multiple proteins, including kelch‐like ECH‐associated protein 1 (KEAP1),^11^ glyceraldehyde 3‐phosphate dehydrogenase (GAPDH),^12^ aldolase A (ALDOA),^13^ receptor‐interacting serine/threonine‐protein kinase 3 (RIPK3),^14^ NLR family pyrin domain containing 3 (NLRP3)^15^ and stimulator of interferon genes (STING).^16^ These investigations have highlighted the potential utility of itaconate or 4‐OI as potential therapeutic targets for a number of inflammatory and infectious diseases. Herein, we reveal the pro‐inflammatory effect of GSDMB‐mediated pyroptosis both in vitro and in vivo, and assess the impact of 4‐OI on pyroptosis, to understand how this lytic cell death is affected in the presence of this metabolic derivative. Our results indicate that 4‐OI blocks GSDMB‐induced pyroptosis by directly targeting granzyme A instead of GSDMB and therefore restricts inflammation.

## 2 RESULTS

### 2.1 GSDMB‐mediated pyroptosis facilitates the pro‐inflammatory immune microenvironment and amplifies inflammation

**FIGURE 1:**
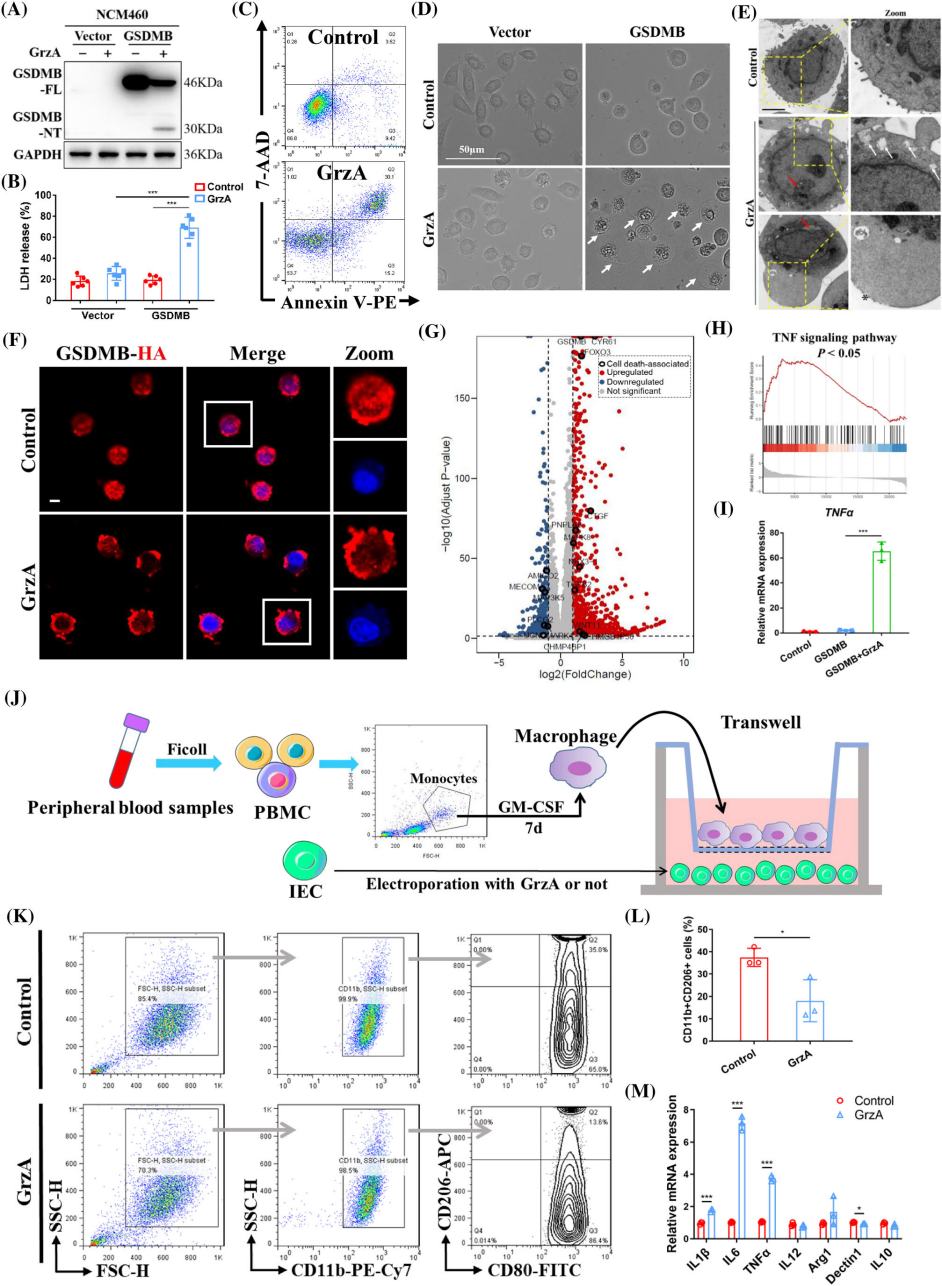
GSDMB mediates pyroptotic cell death of IECs and facilitates the pro‐inflammatory immune microenvironment. (A) Western blot analysis of GSDMB cleavage, (B) LDH release activity from the media, (C) flow cytometry analysis of cell death, (D) representative cell image of pyroptotic ballooning bubbles (white arrows) in GSDMB‐expressing IECs after GrzA electroporation for 6 h. Scale bar, 50 μm. (E) Representative TEM images for pyroptotic morphology of IECs. White arrows showed broken cell membrane, red arrows marked with nuclear envelope breakage, and the asterisk instructed the large bubble. Scale bar = 5 μm. (F) Observation of cellular localisation of GSDMB in IECs. Scale bar, 10 μm. (G) Volcano plots showed the expression of significantly downregulated (blue) and upregulated (red) genes in GSDMB‐expressing IECs after GrzA electroporation compared to that no GrzA treatment. Black circles highlighted genes that involved in the regulation of cell death. (H) Gene Set Enrichment Analysis (GSEA) of TNF signalling pathway. (I) Relative mRNA level of TNFα in IECs. (J) Schematic illustration of GSDMB‐dependent IECs pyroptosis regulated PBMCs‐derived macrophage inflammatory phenotype. (K, L) Flow cytometry analysis for macrophage inflammatory phenotype. (M) The transcript levels of polarisation markers for pro‐ and anti‐inflammatory macrophages by qRT‐PCR. Values were expressed as mean ± SD. *p < 0.05; ***p < 0.001. Data were representative of three independent experiments.

We first investigated whether pyroptosis could indeed occur in intestinal epithelial cells (IECs), as previous studies found that GSDMB did not cause pyroptotic cell death.^17^, ^18^ Similar to other tumour cell lines,^4^ GSDMB was upregulated markedly under stimulation of interferon‐γ (IFN‐γ), and cleaved into the p30 fragment in response to electroporation of purified GrzA in Caco‐2 and HT‐29 cells, the human adenocarcinoma colorectal cell lines, accompanied by significantly increased lactate dehydrogenase (LDH) activity (Supplementary Figure 1A–E). HT‐29 cells developed obvious pyroptotic morphology including leakage of fluorescent dye and formation of large ballooning bubbles due to membrane rupture after being cleaved by GrzA, whereas small interference RNA (siRNA) knockdown of GSDMB inhibited such lytic cell death (Supplementary Figure 1F–H). GSDMB‐induced cell cytotoxicity was also observed in primary IECs (Supplementary Figure 1I–L). To further investigate this hypothesis, GSDMB isoform 3 was reconstituted in NCM460 cells, a human normal intestinal epithelial cell line that lack endogenous expression of GSDMB. GSDMB‐expressing NCM460 cells presented evident lytic death (Figure 1A–C), accompanied by formation of large ballooning bubbles and membrane pore when being cleaved by GrzA (Figure 1D,E). To observe cellular localisation of GSDMB, the HA‐tagged GSDMB was overexpressed in NCM460 cells. GSDMB was found diffusely in the cell under steady state, while the dominant membranous localisation of GSDMB was showed after GrzA electroporation (Figure 1F).

Next we evaluated the pro‐inflammatory impact of GSDMB‐mediated pyroptosis. We found significant enrichment of several biological processes, including regulation of programmed cell death, in GrzA electroporated IECs by gene ontology (GO)‐based functional annotation analysis from RNA‐seq (Supplementary Figure 2A). The cleavage of GSDMB by GrzA led to apparent changes of genes associated with cell death in IECs, such as upregulation of HMGB1, FOXO3 and WNT11 (Figure 1G). KEGG analysis showed that GSDMB cleavage would activate multiple inflammatory pathways, especially TNF signalling pathway (Supplementary Figure 2B and Figure 1H). The mRNA level of *TNFα* also sharply increased in GrzA‐treated GSDMB‐expressing IECs (Figure 1I), suggesting TNFα may be a distinctive inflammatory marker during GSDMB‐induced pyroptosis. By comparison, some other inflammatory genes, such as *IL‐1β*, *IL‐6*, *IL‐11*, *IL‐24* and *CXCL8*, also elevated, but to a lesser extent (Supplementary Figure 2C,D). To further inquire the association between GSDMB‐rendered pyroptosis and inflammation, monocytes‐derived macrophages from health donors, the first line of defence in response to stressors, were co‐cultured with GSDMB‐expressing IECs that electroporated with or without GrzA (Figure 1J). After 2 h of co‐culture, the macrophages were collected, and the results of flow cytometry showed that the proportion of anti‐inflammatory macrophages (CD11b + CD206+) decreased significantly when co‐cultured with IECs treated with GrzA (Figure 1K,L). Accordingly, the transcript levels of polarisation markers for pro‐inflammatory macrophages including IL‐1β, IL‐6 and TNFα were significantly up‐regulated, whereas Dectin‐1, an anti‐inflammatory polarisation marker, was reduced after co‐culture with GrzA‐treated IECs (Figure 1M). These results indicated that a pro‐inflammatory immune microenvironment was shaped in response to GSDMB‐mediated pyroptosis.

### 2.2 Transgenic expression of epithelial GSDMB exacerbates dextran sulphate sodium‐induced inflammation

**FIGURE 2:**
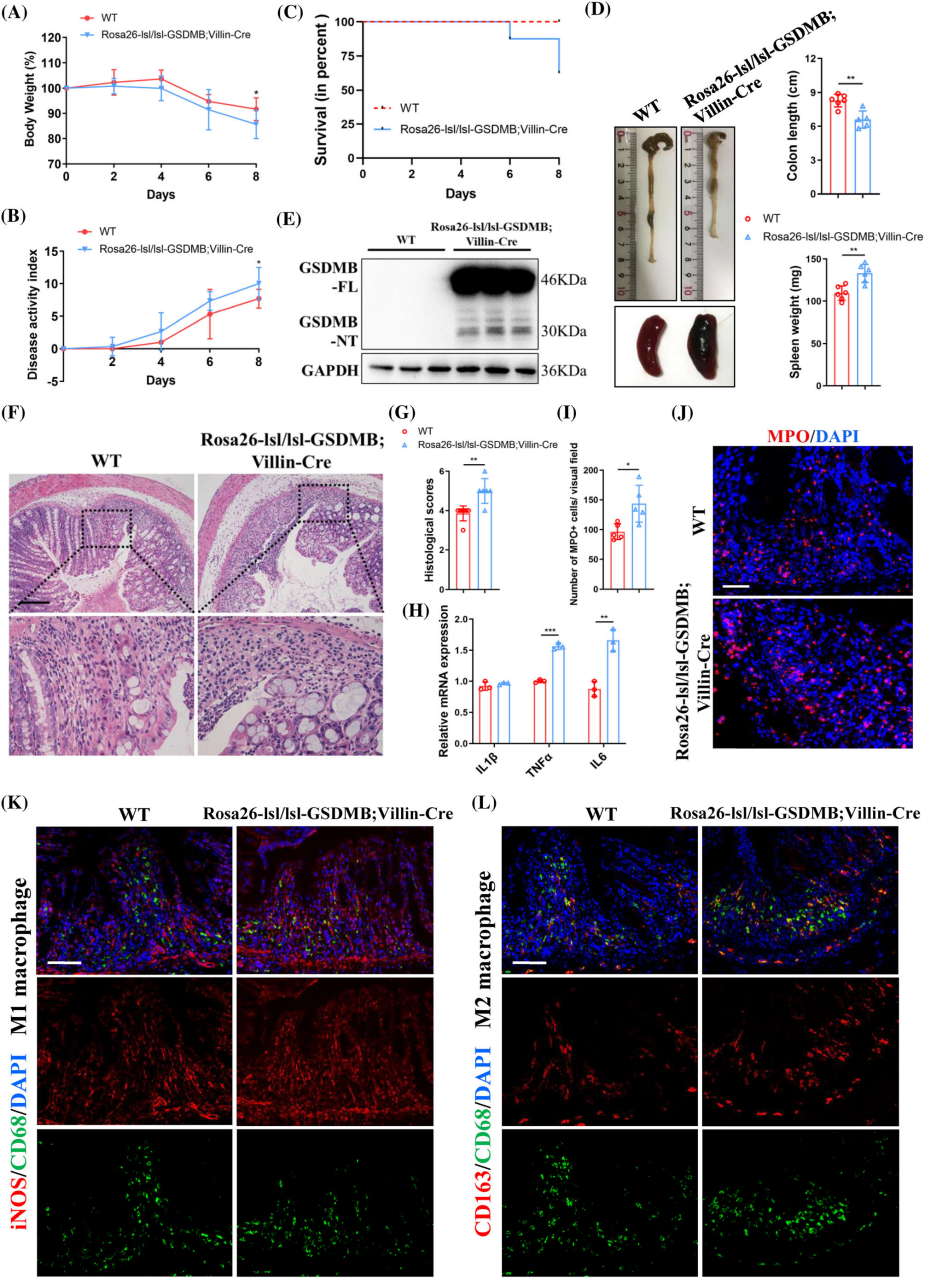
Transgenic expression of epithelial GSDMB leads to increased susceptibility to acute colitis. 8‐week‐old *Rosa26‐lsl/lsl‐GSDMB;Villin‐Cre* and WT mice were administered 2% DSS in drinking water for 7 days to induce acute experimental colitis as indicated (*n* = 5–8/group). (A) Body weight loss, (B) disease activity index, (C) survival rate and (D) gross morphology and length of colon and spleen weight in these two groups. (E) Western blot analysis of GSDMB cleavage in colonic tissues. (F, G) Colonic H&E staining and histological scores after DSS insult. Scale bar, 200 μm. (H) Relative inflammatory cytokine mRNA levels in the colon were measured by qRT‐PCR. (I, J) Representative immunofluorescence staining of MPO in colon of *Rosa26‐lsl/lsl‐GSDMB;Villin‐Cre* and WT mice. Scale bar, 50 μm. (K, L) Representative immunofluorescence staining of iNOS+CD68+ M1 macrophages and CD163 + CD68+ M2 macrophages in colonic tissues. Scale bar, 100 μm. Data were displayed as mean values ± SD. *p < 0.05; **p < 0.01; ***p < 0.001.

IECs generally expose to various luminal stressors including chemicals. To interrogate the functional insight into the effect of GSDMB‐induced pyroptosis in vivo, the chemical stressor dextran sulphate sodium (DSS)‐induced experimental colitis model was employed in the intestinal epithelial GSDMB conditional knock in mice (*Rosa26‐lsl/lsl‐GSDMB;Villin‐Cre*). Strikingly, the clinical signs of colitis, including body weight loss, disease activity index (DAI), mortality, colon shortening and splenomegaly were more severe in *Rosa26‐lsl/lsl‐GSDMB;Villin‐Cre* mice than their WT littermates after one cycle of DSS challenging (Figure 2A–D). In the meantime there was a partial cleavage of GSDMB protein for releasing NT fragment in the colonic tissue of *Rosa26‐lsl/lsl‐GSDMB;Villin‐Cre* mice following DSS insult (Figure 2E). In line with the aggravated disease manifestations, *Rosa26‐lsl/lsl‐GSDMB;Villin‐Cre* mice showed worse mucosal destruction and inflammatory cells infiltration, as well as higher histological score evaluated by haematoxylin–eosin staining (Figure 2F,G). The mRNA expression of inflammatory cytokines TNFα and IL‐6 were also increased in the colon of *Rosa26‐lsl/lsl‐GSDMB;Villin‐Cre* mice (Figure 2H). Correspondingly, more MPO+ neutrophils were observed in the lamina propria of DSS‐induced *Rosa26‐lsl/lsl‐GSDMB;Villin‐Cre* hosts (Figure 2I,J). Moreover, the proportion of M1 macrophages increased while the proportion of M2 macrophages declined in the colonic tissues of *Rosa26‐lsl/lsl‐GSDMB;Villin‐Cre* mice after DSS insult (Figure 2K,L and Supplementary Figure 3A,B), which was consistent with the effect of pyroptosis on macrophages in vitro (Figure 1K–M). Interestingly, no significant difference for IFN‐γ + (Th1), IL‐4+ (Th2), IL‐17A+ (Th17) CD4+ T cells and CD8+ T cells in the mesenteric lymph nodes (MLNs) or lamina propria mononuclear cells (LPMCs) was found between these two groups (Supplementary Figure 3C,D), suggesting the critical role of GSDMB principally in innate immunity during acute colitis. Taken together, these data demonstrated that intestinal expression of GSDMB caused more severe colitis in vivo, at least partially by triggering pyroptosis.

### 2.3 Itaconate and its derivative 4‐OI inhibit GSDMB‐mediated pyroptosis

**FIGURE 3:**
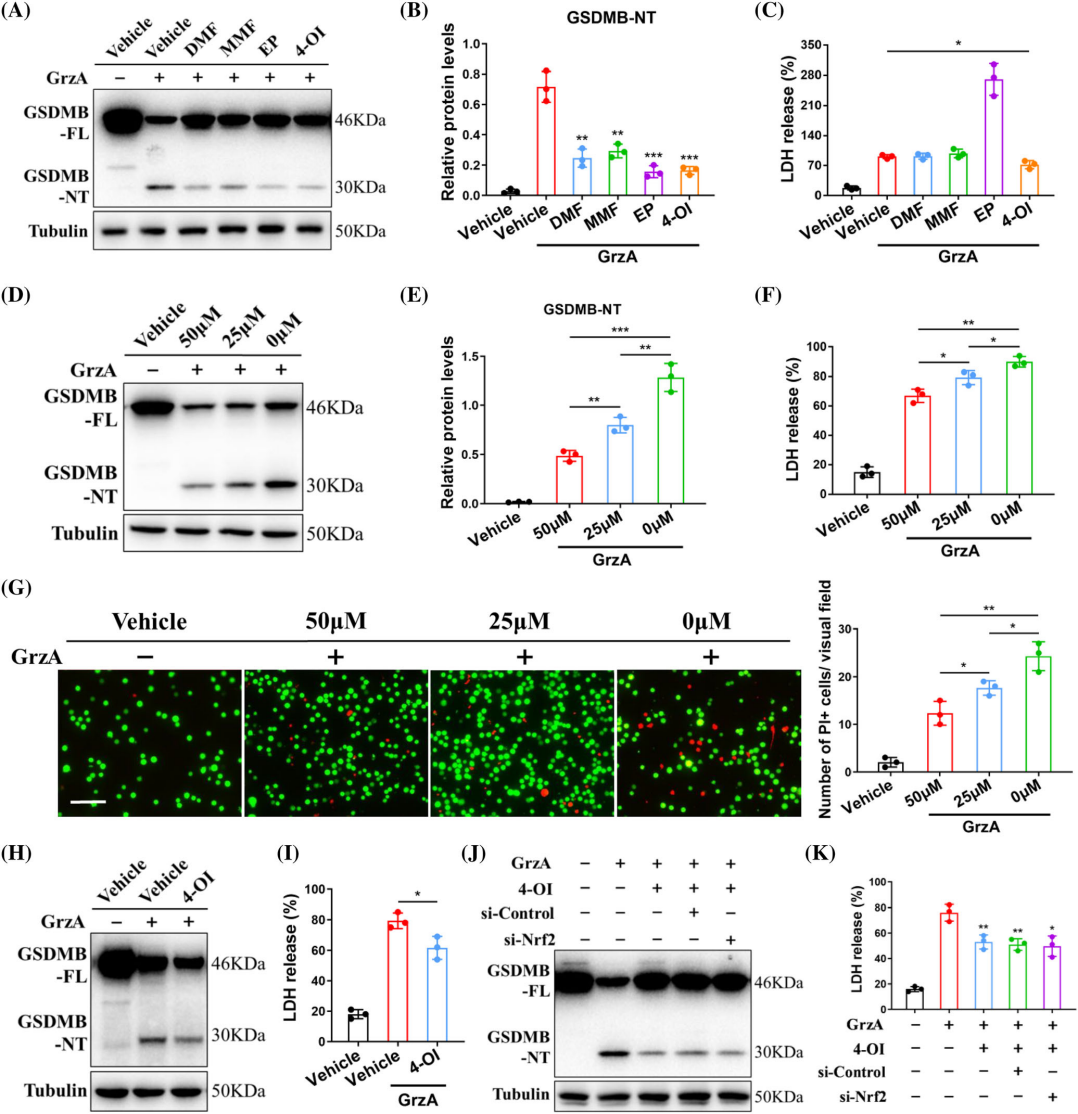
Metabolic derivative 4‐OI suppresses GSDMB‐mediated pyroptosis independent of Nrf2. (A, B) Western blot analysis of GSDMB cleavage in GSDMB‐expressing 293 T cells and (C) LDH release activity from the media when exposed to several metabolites. (D, E) Cleavage of GSDMB and (F) LDH release activity in GSDMB‐expressing 293 T cells when exposed to different concentrations of 4‐OI. (G) Representative image of live/dead assay in GSDMB‐expressing 293 T cells after electroporated with GrzA when exposed to different concentrations of 4‐OI. Live cell (green), Calcein AM. Dead cell (red), PI. Scale bar, 100 μm. Shown on the right was the number of PI+ cells. (H) Cleavage of GSDMB and (I) LDH release activity in human primary IECs after 4‐OI treatment. (J) Western blot analysis of GSDMB cleavage and (K) LDH release activity when transfected with siRNA of Nrf2. Data were displayed as mean values ± SD. *p < 0.05; **p < 0.01; ***p < 0.001.

Considering that GSDMB‐induced pyroptosis amplifies inflammation dramatically, it is necessary to look for effective strategy to suppress the pro‐inflammatory functions of GSDMB. It was reported that Krebs cycle intermediates such as itaconate and succinate accumulated and served as regulators of inflammatory gene expression.^19^ Therefore, we investigated whether Krebs cycle intermediates could modulate functions of GSDMB. The GSDMB‐expressing 293 T cells were electroporated with GrzA and then exposed to several metabolites for 6 h. The occurrence of pyroptosis was measured by GSDMB cleavage and LDH release. Interestingly, the cell‐permeable itaconate, 4‐OI simultaneously blocked the formation of GSDMB‐NT and suppressed release of LDH (Figure 3A–C). Other synthetic metabolic derivatives, such as dimethyl fumarate (DMF), monomethyl fumarate (MMF) and ethyl pyruvate (EP), although lessened cleavage of GSDMB to some extent, had little effect on reducing LDH release, indicating the potential of 4‐OI to inhibit GSDMB‐rendered pyroptosis. Strikingly, the pyroptosis was suppressed in a dose‐dependent manner by 4‐OI treatment in GSDMB‐expressing 293 T cells (Figure 3D–G). Furthermore, 4‐OI was able to decrease GSDMB‐induced pyroptosis in human primary IECs (Figure 3H,I and Supplementary Figure 4A).

It was demonstrated that 4‐OI exerted anti‐inflammatory effect by Nrf2, an antioxidant nuclear transcription factor that senses oxidative and electrophilic stress.^20^ Therefore we identified 4‐OI inhibited pyroptotic cell death whether required Nrf2. The small interfering RNA for Nrf2 was constructed and transfected before GrzA treatment. Surprisingly, 4‐OI could still restrain GSDMB‐mediated pyroptosis obviously after Nrf2 knockdown (Figure 3J,K and Supplementary Figure 4B), suggesting an unnecessary role of Nrf2 during this anti‐pyroptotic process. Next we evaluated whether endogenous itaconate can cause the same effect on pyroptosis. We found that overexpression of a plasmid encoding IRG1 also resulted in reduced GSDMB cleavage and LDH release in response to GrzA treatment (Supplementary Figure 4C–E). Collectively, these results suggested 4‐OI and itaconate were capable of inhibiting GSDMB‐induced pyroptosis independent of Nrf2.

### 2.4 4‐OI inhibits pyroptosis not through interaction with GSDMB

A few studies have identified several small‐molecule inhibitors that repress GSDMD‐related pore formation and lytic cell death by directly binding GSDMD, such as disulfiram, necrosulfonamide and DMF.^21^, ^22^, ^23^ Therefore we hypothesised that 4‐OI may inhibit GSDMB‐mediated pyroptosis by binding GSDMB. To verify this hypothesis, we detected GSDMB‐induced pyroptotic cell death for exposing to 4‐OI in different phases. Unexpectedly, there was almost no difference for pyroptosis when GSDMB‐expressing 293 T cells were pre‐treated with 4‐OI before GrzA electroporation (Supplementary Figure 5A–C). In comparison, the GSDMB‐NT formation and LDH release were restrained when treated with 4‐OI after GrzA electroporation or treated with 4‐OI concurrently before and after GrzA electroporation, indicating that 4‐OI might disturb GrzA‐related process rather than directly inhibiting GSDMB. To further validate our conjecture, the in vitro incubation of GrzA with 4‐OI was performed. GSDMB‐expressing 293 T cells had decreased GSDMB oligomerisation in response to electroporation with GrzA that premixed with 4‐OI immediately, while the cleavage of GSDMB and its oligomerisation were almost completely impeded when electroporated with GrzA that preincubated with 4‐OI for 1 h (Supplementary Figure 5D,E). A notable reduction of LDH release was also observed after electroporation with GrzA that preincubated for 1 h with 4‐OI (Supplementary Figure 5F). Consistently, the membranous localisation of GSDMB was also impeded when GrzA premixed with 4‐OI for 1 h (Supplementary Figure 5G). Importantly, co‐incubation with 4‐OI did not degradation of the recombinant GrzA protein (Supplementary Figure 5H). These data implied that 4‐OI inhibited pyroptosis by interacting with GrzA instead of GSDMB.

### 2.5 4‐OI directly targets Granzyme A to block pyroptosis

**FIGURE 4:**
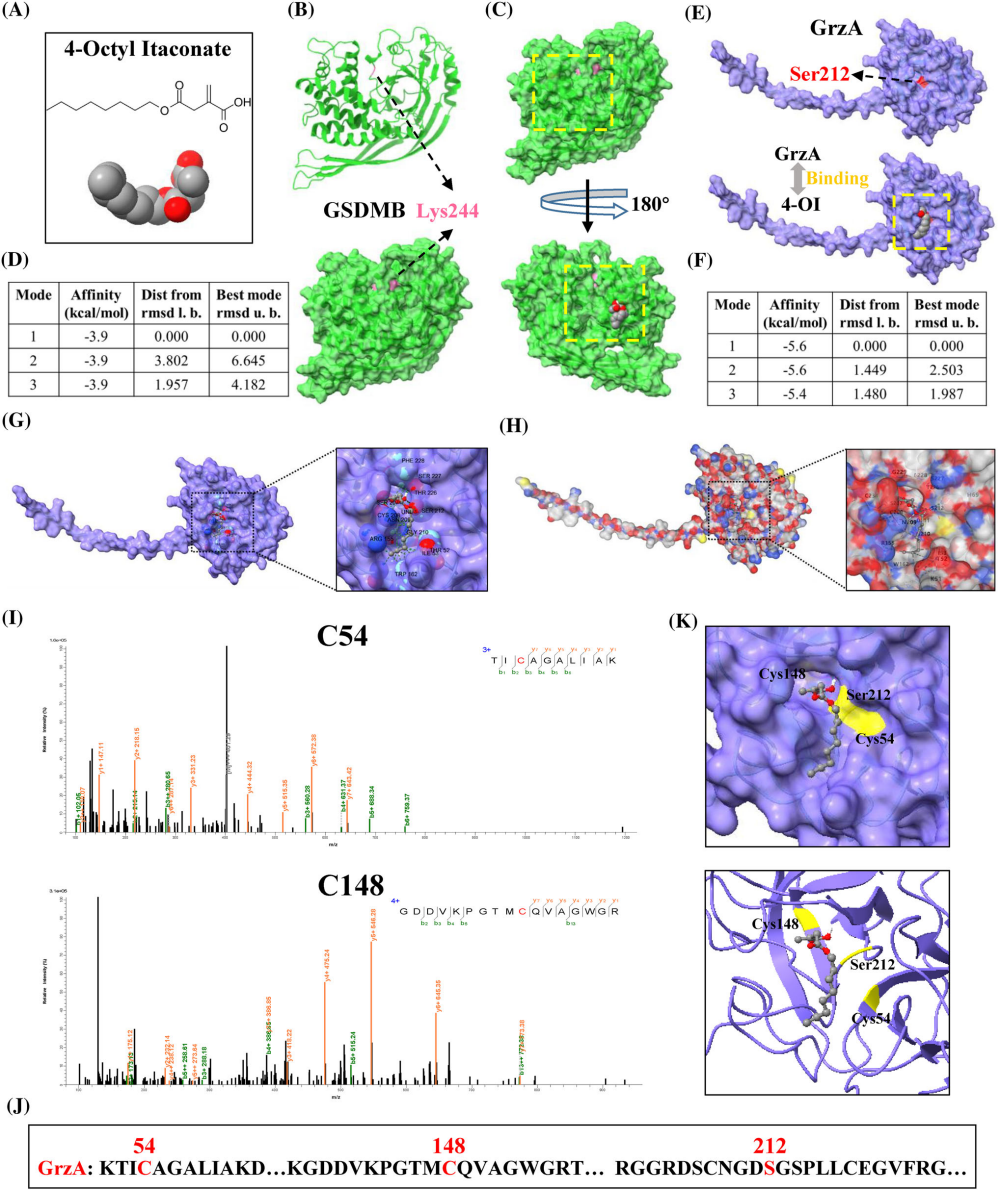
4‐OI directly modifies Granzyme A to block GSDMB‐rendered pyroptosis. (A) Chemical and molecular structure of 4‐OI. (B) Molecular structure of GSDMB and its major cleavage site Lys244 by GrzA. (C, D) Molecular docking program AutoDock Vina predicting the possible interaction between 4‐OI and GSDMB. (E, F) Molecular structure of GrzA and its enzymatic Ser212 residue that cleaving GSDMB, and the possible interaction between 4‐OI and GrzA. (G, H) Amino acid residues visualisation on GrzA that interacted with the atoms of 4‐OI in two molecular docking program AutoDock Vina and CB‐Dock2, respectively. (I) Tandem mass spectrometry spectrum of GrzA peptide following 4‐OI treatment (0.5 mM, 6 h). (J) Amino acid sequences of human GrzA, with identified modification sites in red. (K) Spatial position relation between 4‐OI and identified modification sites Cys54, Cys148 and Ser212 on GrzA. Data were representative of three independent experiments.

To further confirm the targeting effect of 4‐OI on GrzA, we employed two molecular docking program AutoDock Vina and CB‐Dock2 to predict the possible interaction between 4‐OI and GSDMB or GrzA. The structure of GSDMB and GrzA were downloaded from RoseTTAFold and AlphaFold protein structure database. According to the docking results of AutoDock Vina, 4‐OI was unlikely to interact with GSDMB due to the relatively low affinity and high root mean square deviation (RMSD) values; even if it interacted, it would bind to C‐terminal domain of GSDMB, which did not affect the major cleavage site Lys244 by GrzA or pore forming ability of NT domain of GSDMB in spatial position (Figure 4A–D). Howbeit a highly possible combination between 4‐OI and GrzA was predicted as shown by high affinity and low RMSD (Figure 4E,F). From the docking model, we observed that 4‐OI could be embedded into the recessed domain of GrzA, which just right blocked the enzymatic Ser212 residue in GrzA that cleaving GSDMB. Further visualisation of the interaction between the atoms of 4‐OI and the amino acid residues of GrzA revealed a significant binding between 4‐OI and numerous residues of GrzA including Ser212 (Figure 4G). Since a previous study claimed that GrzA formed a stable disulphide‐linked homodimer to trigger target‐cell apoptosis,^24^ we examined the possibility of 4‐OI hindering the formation of dimer. It seemed unlikely to prohibit the formation of GrzA homodimer due to the high RMSD values (Supplementary Figure 6A–C). Similarly, the results of another protein‐ligand docking tool CB‐Dock2 also showed a highly probable binding between 4‐OI and GrzA (Supplementary Figure 6D). This model predicted 4‐OI would insert into the concaved structural domain of GrzA, and bind to multiple amino acid residues in GrzA, including Ser212. (Supplementary Figure 6E and Figure 4H).

Next, liquid chromatography–tandem mass spectrometry (LC–MS/MS) of human GrzA, immunoprecipitated from 4‐OI treated GrzA‐overexpressing 293 T cells, was performed. LC–MS/MS analysis showed that 4‐OI modified GrzA directly and this itaconation (+242.15 Da) occurred at Cys54, Cys148 and Ser212 residues on human GrzA (Figure 4I,J and Supplementary Figure 6F). These three amino acid residues were just located in the groove structure of GrzA where 4‐OI was embedded (Figure 4K), and most importantly, Ser212 was the key amino acid site of GrzA to cleave GSDMB. These data implied that 4‐OI interfered proteolytic cleavage process of GSDMB by directly modifying Cys54, Cys148 and Ser212 on GrzA, thereby blocking pyroptosis.

### 2.6 4‐OI reduces pyroptosis and restricts inflammation in vivo

**FIGURE 5:**
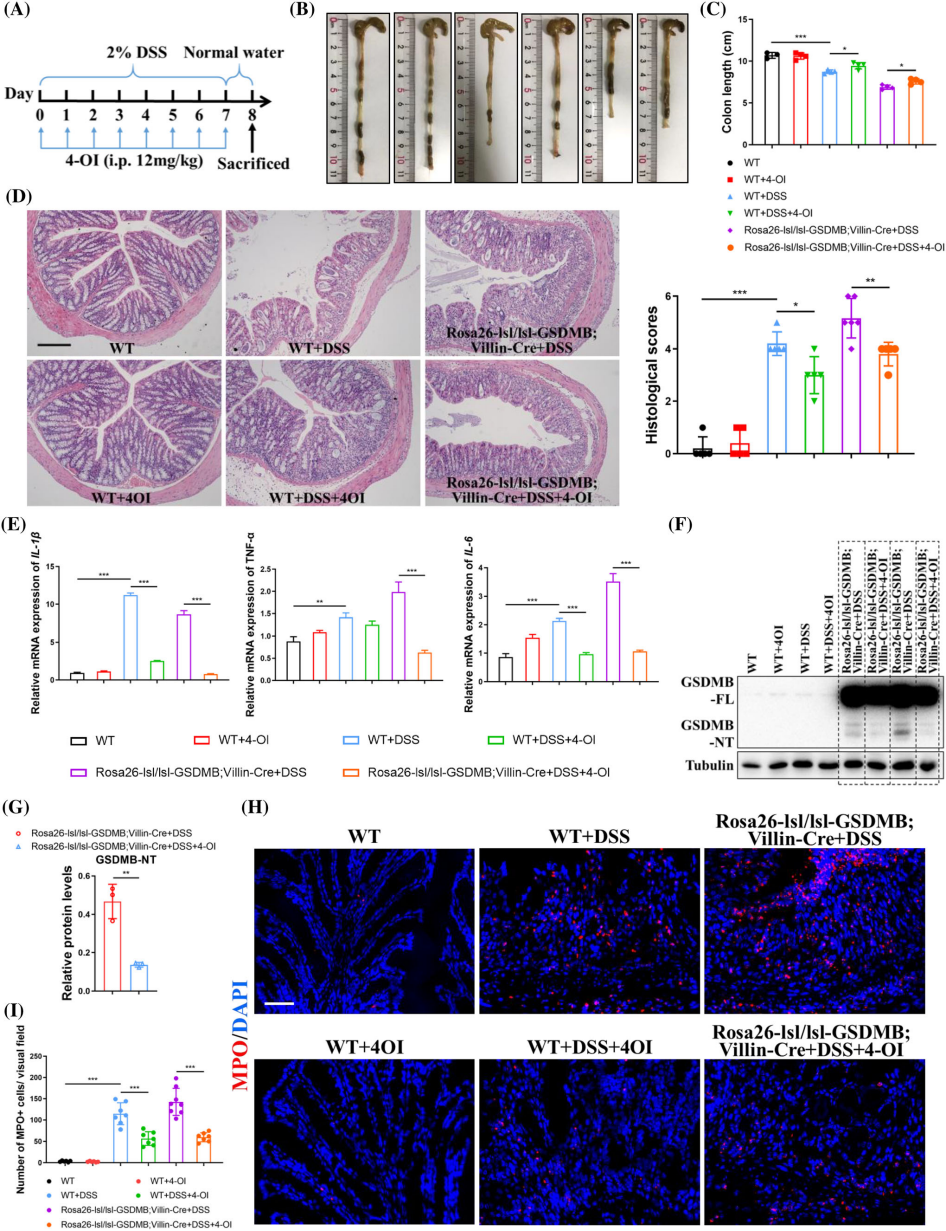
4‐OI reduces pyroptosis and restricts intestinal inflammation in vivo. (A) The experimental scheme for the administration of 4‐OI during DSS‐induced experimental colitis. The 4‐OI was intraperitoneally injected daily at 12 mg/kg for 7 days (*n* = 4 to 8 per group). (B, C) Gross morphology and length of colon in different groups. (D) Representative H&E staining of colon sections after DSS insult. Scale bar, 200 μm. Shown on the right was the histological scores. (E) Relative inflammatory cytokine mRNA levels in the colon that measured by qRT‐PCR. (F, G) Blocking effect of 4‐OI on proteolytic cleavage of GSDMB in colonic tissues of WT and *Rosa26‐lsl/lsl‐GSDMB;Villin‐Cre* colitis mice by western blot. (H, I) Representative immunofluorescence staining of MPO in the colon of colitis mice. Scale bar, 50 μm. Data were displayed as mean values ± SD. *p < 0.05; **p < 0.01; ***p < 0.001.

Since 4‐OI suppressed GSDMB‐dependent pyroptosis, we next evaluated the anti‐inflammatory effect of 4‐OI on experimental colitis in vivo. In the mild DSS‐induced acute colitis model, 4‐OI was intraperitoneally injected daily for 7 days (Figure 5A). Intriguingly, not only WT mice but also *Rosa26‐lsl/lsl‐GSDMB;Villin‐Cre* mice treated with 4‐OI exhibited partially recovered colon shortening after DSS insult (Figure 5B,C). The histological inflammation and inflammatory cytokines mRNA expression induced by DSS were also partially recovered by 4‐OI treatment (Figure 5D,E). Correspondingly, western blot analysis of colonic tissues confirmed that 4‐OI was able to block proteolytic cleavage of GSDMB in *Rosa26‐lsl/lsl‐GSDMB;Villin‐Cre* colitis mice (Figure 5F,G). Furthermore, the MPO activity decreased a lot when mice were treated with 4‐OI (Figure 5H,I). These results indicated that metabolic derivative 4‐OI suppressed GSDMB‐mediated pyroptotic cell death and mitigated intestinal inflammation, emphasising a therapeutic effect of exogenous supplementation of 4‐OI.

## 3 DISCUSSION

Our data demonstrated that metabolic derivative 4‐OI inhibited GSDMB‐mediated pyroptosis by directly binding to GrzA via modification of Cys54, Cys148 and Ser212, identifying another mechanism for the immunomodulatory and anti‐inflammatory effects of 4‐OI. It is generally accepted that pyroptosis executed by GSDMs plays a critical role in the pathogenesis of several primarily inflammatory and/or autoimmune diseases. Pyroptosis is a pro‐inflammatory form of programmed cell death that provoked by stimuli including damage‐associated molecular patterns (DAMPs) such as stress signals, oxidised lipoproteins, or pathogen‐associated molecular patterns (PAMPs) such as flagellin, lipopolysaccharide (LPS) and type 3 secretion system (T3SS) structure proteins.^25^ Upon pyroptotic cell death against cell stress/danger signal or pathogen invasion, a plethora of inflammatory cellular contents are released and trigger a cascade of immune responses from the neighbouring cells.^26^ Overactivated pyroptosis may lead to tissue damage, and sometimes organ failure and even host death. Our recent studies have uncovered the function and mechanism of GSDMD‐induced pyroptosis during intestinal inflammation and its associated colorectal cancer.^27^, ^28^ And here, we confirmed that GSDMB‐induced pyroptosis in IECs enhanced the pro‐inflammatory microenvironment, thereby amplifying intestinal inflammation. The pyroptosis caused by GSDMB cleavage in vivo was relatively weak, mainly because murine GrzA cleaved the human GSDMB protein that overexpressed in *Rosa26‐lsl/lsl‐GSDMB;Villin‐Cre* mice. Although it can cleave, its efficiency is significantly reduced compared to homologous human GrzA.^4^ That is to say, such non‐homology led to a small amount of GSDMB‐NT, which was difficult to avoid in vivo experiments due to the lack of a mouse GSDMB orthologue. Interestingly, the previous study reported GSDMB enhanced proliferation and migration of IECs by regulating focal adhesion kinase (FAK) phosphorylation rather than its driven pyroptosis, thus promoting restoration of epithelial barrier function and the resolution of inflammation.^18^ Indeed, GSDMB isoforms produced by alternative splicing have full or no pyroptotic activity, and only GSDMB isoforms 3, 4 and 6 containing exon 6 dictate lytic activity. Therefore, the lack of inherent GSDMB‐dependent pyroptosis in activated IECs in the study of Rana N et al.^18^ was due to the use of non‐canonical splicing isoform of GSDMB, whereas isoforms 3 was employed in our study, which has a widely prominent expression in tissues and the strongest ability to induce pyroptosis.^5^, ^7^, ^8^

Appropriate inflammation facilitates the activation of the innate immune system against stimuli, while excessive inflammatory responses are detrimental and even lethal. Therefore, it is necessary to seek effective strategy to suppress overactivated inflammation caused by pyroptosis in certain diseases. In our study, we found that 4‐OI significantly alleviated experimental colitis by inhibiting GSDMB‐mediated pyroptosis, at least in part. Previous studies pointed that IRG1 deletion resulted in substantial release of inflammatory mediators and aggravation of DSS‐induced colitis, and the disease condition was greatly improved by exogenous supplementation with 4‐OI.^29^, ^30^ However, the exact mechanism of the effects of 4‐OI in the context of colitis is largely unknown. Our work reveals the beneficial effects of 4‐OI from another perspective, that is, 4‐OI can directly modify Cys54, Cys148 and Ser212 on GrzA to abolish its cleavage ability to GSDMB, further enriching the anti‐inflammatory mechanistic basis of 4‐OI. Moreover, we noticed that the use of 4‐OI in GSDMB deficient mice (WT mice) also relieved intestinal inflammation, indicating that 4‐OI could conduct some other functions apart from such pyroptosis to contribute to anti‐inflammatory effects. Indeed, 4‐OI alkylated Cys151, 257, 288, 273 and 297 on the protein KEAP1 to activate Nrf2 and to exert the anti‐oxidant and anti‐inflammatory capacities.^11^ Our recent study confirmed that 4‐OI alkylation modified Cys147 of STING to down‐regulate inflammation.^16^ It was also found that itaconate and 4‐OI modified NLRP3 at Cys548 and GSDMD at Cys77,^15^, ^31^ adding further weight to the concept of the inhibitory effect for 4‐OI on pyroptosis. These studies increase the evidence that itaconate, with profound anti‐inflammatory effects, is a critical determinant of innate immune responses.

Our work employed 4‐OI, a considered proxy of itaconate, in the experiments due to the low cell permeability of itaconate. It was demonstrated that ^13^C_5_‐labelled octyl itaconate was converted into ^13^C_5_ itaconate intracellularly and there was significant overlap in the cysteine residues alkylated by 4‐OI and endogenous itaconate.^11^ Furthermore, the relative electrophilicity of 4‐OI resulted in a higher ability to affect certain pathways compared to that of unmodified itaconate.^32^ Our results also validated a comparable anti‐pyroptotic effect between endogenous itaconate and 4‐OI. The above findings make 4‐OI a suitable itaconate substitute for studying its biological function.

Overall, our work provide evidence that 4‐OI, as an inflammatory regulator, directly inhibits pyroptotic activity through a newly identified posttranslational modification and thereby exerts anti‐inflammatory effects. The targeting of pyroptosis by 4‐OI therefore holds potent therapeutic potential for inflammatory diseases and expands the role of 4‐OI as a crucial immunometabolic derivative that regulates innate immunity and inflammation.

## 4 MATERIALS AND METHODS

### 4.1 Mice

All mice on a C57BL/6J background were used in this study. The human GSDMB targeting plasmid construct with mRosa26‐pCAG‐loxp‐mcherry‐stop‐loxp‐hGSDMB‐BP was generated by cloning the 1248‐bp hGSDMB ORF (which represents the entire length of the human GSDMB gene, namely GSDMB isoform 3) from pCAG‐loxp‐mcherry‐stop‐loxp‐BPA with AgeI/NotI into a construct provided by Jiangsu Laboratory Animal Center at Nanjing Medical University. SpeI/SacI/CIAP‐linearised mRosa26‐pCAG‐loxp‐mcherry‐stop‐loxp‐hGSDMB‐BP was microinjected into the pronuclei of fertilised mouse embryos and implanted into a C57BL/6J background‐pseudopregnant mouse. The transgene construct has a loxP‐flanked mcherry and transcriptional stop codon positioned at the transcriptional initiation site of the human GSDMB transgene, which prevented transcription of GSDMB. The GSDMB floxed mice with mRosa26‐pCAG‐loxp‐mcherry‐stop‐loxp‐hGSDMB‐BP transgene construct (*Rosa26‐lsl/lsl‐GSDMB*) were crossed with *Villin*‐Cre mice to generate IECs‐specific *GSDMB*‐knock in mice (homozygote: *Rosa26‐lsl/lsl‐GSDMB;Villin‐Cre*). In vivo models were performed with 6 to 8‐week old male mice and littermates were randomly assigned to experimental groups. All of the mice were maintained on a 12 h‐dark/light cycle with the temperature of 20–26°C and 30%–40% humidity under specific pathogen‐free conditions. All animal procedures were ethically approved by the Biomedical Ethics Committee of Health Science Center of Xi'an Jiaotong University (No. XJTUAE2023‐300) and the Institutional Animal Care and Use Committee of Nanjing Medical University (No. IACUC‐2109035).

### 4.2 DSS‐induced colitis model

The experimental colitis model was induced by using DSS. Briefly, mice received 2% (w/v) DSS (M.W. 36,000–50,000, MP Biomedicals) in drinking water for 7 days. The weight of mice was observed at the same time point every day. Disease activity index (DAI) was calculated daily based on weight loss, stool consistency, and the degree of intestinal bleeding. The mice were euthanised at the end of day 8, and the colon lengths were recorded and colonic tissues were collected. In selective experiments, mice were treated with 4‐OI (12 mg/kg) intraperitoneally every day during DSS insult.

### 4.3 Cell culture

Caco‐2 and HEK293T cells were grown in Dulbecco's modified Eagle's medium (DMEM) supplemented with 10% (v/v) foetal bovine serum (FBS), 2 mM L‐glutamine and 2% penicillin/streptomycin. HT‐29 cells were cultured in McCoy's 5A medium modified with l‐glutamine and sodium bicarbonate, supplemented with 10% FBS and 2% penicillin/streptomycin. NCM460 cells and primary IECs were grown in RPMI 1640 medium containing 10% FBS, 2 mM l‐glutamine and 2% penicillin/streptomycin. Cell lines were authenticated by STR profiling and tested to be mycoplasma‐negative by the standard PCR method.

In selective experiments, indicated cells were cultured in medium containing dimethyl fumarate (25 μM), monomethyl fumarate (25 μM), ethyl pyruvate (5 mM) and 4‐octyl itaconate (50 μM) for 1 h, respectively. For the in vitro 4‐OI reaction premixing with GrzA, 1 μg of recombinant human GrzA was incubated with 50 μM of 4‐OI for 1 h at room temperature and then electroporated into GSDMB‐expressing 293 T cells.

### 4.4 Generation of human monocytes‐derived macrophages

First, peripheral blood mononuclear cells (PBMCs) were isolated from whole blood of health consenting donors. Blood was diluted 1:1 in sterile PBS and layered over the Lymphoprep with a volume of two times. Blood was spun at 500 g with no break. The interphase was transferred to a fresh tube and washed twice in PBS. Following lysing in red blood cell lysis buffer for 10 min at room temperature to remove red blood cells, cells were washed twice in PBS and counted.

Next, the monocytes were sorted from the PBMCs by flow cytometry and were incubated with 5 ng/mL GM‐CSF for 7 days to generate macrophages. The GM‐CSF differentiated macrophages were co‐cultured with IECs that electroporated with GrzA or not for 2 h in a transwell system. Differentiated macrophages were placed into the upper chamber and IECs were placed in the bottom chamber.

### 4.5 Isolation of human primary intestinal epithelial cells

The human intestine tissues taken during intestinal surgery were washed with Hank's balanced salt solution (HBSS). After washing, the tissues were cut into small pieces and shaken in RPMI 1640 medium containing 5% FBS, 1.5 mM EDTA and 10 mM dithiothreitol for 30 min at 37°C. The remaining tissue fragments were removed, and epithelial cells in the supernatant were spun down at 150 g for 5 min.

The collection of human specimens was approved by the institutional review board at the First Affiliated Hospital of Xi'an Jiaotong University (No. XJTU1AF2023LSK‐505), and each participant provided written informed consent.

### 4.6 Isolation of mouse lamina propria mononuclear cells

After removing the IECs, the remaining intestinal tissues were digested with 25 mmol/L HEPES buffer containing 0.02% collagenase and 0.01% DNaseI at 37°C with shaking for 30 min. Lamina propria cells were collected after passing through a 70 μm strainer and then purified via density gradient centrifugation with 40% and 70% Percoll‐RPMI solution. LPMCs were obtained from the interphase.

### 4.7 Plasmids and transfection

The DNA fragments encoding GSDMB (isoform 3) were amplified from human genomic complementary DNA (cDNA) and inserted into the expressing vector pCMV3‐untagged following by DNA sequencing. GSDMB plasmid was transfected into 293 T cells using lipofectamin 3000 reagent (Invitrogen, L3000008) and into NCM460 and primary IECs using the 4D‐NucleofectorTM electroporation system (Lonza) following the manufacturer's instructions. The transfection reagent SF Cell Line 4D‐NucleofectorTM X Kit L (Lonza, V4XC‐2024) and P3 Primary Cell 4D‐Nucleofector X Kit L (Lonza, V4XP‐3024) were used.

To induce pyroptosis in vitro, 1 μg of recombinant human GrzA protein (BioVision, USA) was electroporated into indicated cells using the 4D‐NucleofectorTM electroporation system (Lonza). After 6 h of electroporation, samples were collected and LDH release in the supernatants was measured.

### 4.8 LDH assay

LDH release was analysed using LDH Cytotoxicity Assay as per manufacturer's protocol. Percent cell death was calculated by LDH release = (sample‐untreated)/(maximum‐untreated) × 100.

### 4.9 Western blotting

Cells and mouse intestinal tissues were homogenised in RIPA buffer containing protease inhibitor cocktail and heated at 95°C for 5 min. For the GSDMB oligomer assay, supernatants were mixed with a loading buffer that lacked β‐mercaptoethanol. SDS‐polyacrylamide gel electrophoresis (PAGE) gels were used to separate the protein samples and transferred onto a polyvinylidene fluoride membrane via wet transfer.

### 4.10 Flow cytometry

Samples were run on a FACS Calibur (BD Bioscience) and analysed with FlowJo software (Treestar Inc). After removing the media supernatant, macrophages were washed with PBS and then removed using a cell scraper. Macrophages, LPMCs and lymphocytes from mesenteric lymph nodes were treated with Fcγ‐blocking antibody anti‐human BD Fc block (eBioscience, 564,219) or anti‐mouse CD16/32 (eBioscience, 553141) for 10 min before being stained with fluorochrome‐conjugated antibodies. Directly conjugated antibodies used for surface or intracellular staining were as follows: CD11b (eBioscience, 25–0113‐41), CD80 (eBioscience, 11–0809‐41), CD206 (eBioscience, 17–2069‐41), Th1/Th2/Th17 phenotyping cocktail (eBioscience, 51–9,006,631), CD4 (eBioscience, 553,046), CD8 (eBioscience, 11–0081‐82). Cell death was analysed by using the Annexin V‐PE/7‐AAD Cell Apoptosis Detection Kit (Servicebio, G1512) according to the manufacturer's protocol.

### 4.11 Transmission electron microscopy

The IECs electroporated with or without GrzA were collected and immersed in 2.5% glutaraldehyde, following fixing in 1% osmium tetroxide. After dehydrating in an ethanol gradient and embedding in araldite, the thin sections were stained with uranyl acetate and lead citrate, and then scanned using an H‐7650C transmission electron microscope (Hitachi, Japan) at 80 kV.

### 4.12 RNA isolation and real‐time quantitative PCR

The transcription levels of tissues and cells were determined by quantitative real‐time PCR. Briefly, total RNA was extracted with a TRIzol reagent (Invitrogen, Carlsbad, CA, USA) and reverse‐transcribed with HiScript III RT SuperMix. Afterwards, 1 μL of template was added in a 10‐μL reaction containing 1.0 μL of each primer and 5 μL of SYBR Green QPCR Master Mix. Results were normalised with β‐actin and shown as relative expression value using the ΔΔCt method.

### 4.13 RNA sequencing

Profiling of transcriptome analysis was performed with 2 μg high‐quality total RNA per sample by RNA‐sequencing at Annoroad Gene Technology Corporation (Beijing, China). Detailed procedures were described previously.^28^ Raw sequencing reads were cleaned by removing adaptor sequences, low‐quality reads and reads containing poly‐N sequences, and clean reads were then mapped to GenBank to identify known human mRNA. The original RNA‐seq data were deposited in the NCBI Sequence Read Archive under accession number SRR23071386.

### 4.14 Histological analysis

Fresh colonic samples were fixed in 4% paraformaldehyde for histological staining. The intestinal tissues were dried in ethanol, embedded in paraffin, and stained with haematoxylin and eosin (H&E). The scoring system for inflammation‐associated histological changes of the intestinal samples contained two histological components, which are tissue damage and lamina propria inflammatory cell infiltration. The sum of these two subscores resulted in a total score ranging from 0 (no changes) to 6 (widespread cellular infiltrations and extensive tissue damage).

### 4.15 Immunofluorescence staining

Cells were seeded on poly‐l‐lysine‐coated coverslips, subjected to a single PBS wash, fixed with 4% paraformaldehyde and permeabilised with 100% ice‐cold methanol. After blocking with 5% BSA for 1 h, the coverslips were stained with primary antibodies overnight at 4°C. Tissue sections were incubated at 4°C overnight with primary antibody and then incubated with indicated secondary antibodies. The nuclei were counterstained with DAPI.

### 4.16 RNA interference

The double‐stranded siRNA targeting human *GSDMB* and *Nrf2* were synthesised by General Biosystems (Anhui, China) Co. Ltd. The siRNA sequence of *GSDMB* and *Nrf2* were 5′‐GCUGUAUGUUGUUGUCUCUAUTT‐3′ and 5′‐GAGUAUGAGCUGGAAAAACUU‐3′, respectively. For siRNA knockdown, 50 pmol of siRNAs were transfected into HT29 cells and HEK293T cells using Lipofectamine 3000 reagent (Invitrogen). After 72 h of the transfection, the samples were analysed by immunoblotting to validate the siRNA knockdown efficiency.

### 4.17 In silico protein prediction tools

The structure of GSDMB and GrzA were downloaded from RoseTTAFold (https://robetta.bakerlab.org/) and AlphaFold (www.alphafold.ebi.ac.uk/entry/P12544) protein structure database, respectively. The possible interaction between 4‐OI and GrzA or GSDMB was deduced using AutoDock Vina (https://vina.scripps.edu/) and CB‐Dock2 online webserver (https://cadd.labshare.cn/cb-dock2/php/index.php), which both allow to predict the interacting residues. These two models allow selecting the possible interaction according to the predicted affinity and Vina score. The structure with the higher affinity accompanied by the least RMSD or the highest Vina score was selected.

### 4.18 Analysis of GrzA modification by 4‐OI

HEK293T cells that transfected with plasmid encoding human FLAG‐Granzyme A (Sino Biological, HG13325‐NF) using lipofectamine 3000 for 24 h were treated with 4‐OI (0.5 mM) for 6 h. Following treatment, cells were lysed in lysis buffer and immunoprecipitated with anti‐FLAG antibody and protein A/G agarose beads. Next the beads were washed three times with 1 mL lysis buffer after centrifuging at 400 g for 2 min. The immune complexes were eluted by addition of 40 mL lysis buffer, dissolved in 1x SDS sample buffer and boiled for 5 min. The samples were separate by SDS‐PAGE and subsequently probed with Coomassie blue. The corresponding bands for FLAG‐Granzyme A were excised from the gel and subjected to in‐gel digest with trypsin. Briefly, the gel slices were cut into smaller pieces before reduction with dithiothreitol (10 mM) and then dehydrated in 100% acetonitrile. Gel slices were digested with trypsin overnight at 37°C. Peptides were eluted from the gel pieces following protease digest and dried down completely in a vacuum centrifuge. Samples were analysed in a Q Exactive TM Plus (Thermo Fisher) coupled online to UPLC after the nanospray ionisation source. MS data was analysed with Proteome Discoverer 1.3 and tandem mass spectra were searched against the UniProt database. Trypsin/P is designated as a cleavage enzyme that allows up to two deletion cleavage. Precursor mass tolerance was set to 10 ppm, while fragments were detected with a tolerance of 0.02 Da.

### 4.19 Quantification and statistical analysis

Statistical analyses were carried out using GraphPad Prism 9 (GraphPad) software. All data were presented as mean ± standard deviation or as individual data points. The two‐way Student's *t* test was used to compare two groups affected by a single variable, while a one‐ or two‐way ANOVA was used to compare multiple groups. Significance was defined as follows: *p < 0.05; **p < 0.01; ***p < 0.001.

## AUTHOR CONTRIBUTIONS

WG, TZ, KG and WZ conceived the study and analysed the data; WG, KY and HF conducted the research and collected the data; WG and WZ wrote and edited the manuscript. All authors read and approved manuscript.

## FUNDING INFORMATION

This work was supported by the National Natural Science Foundation of China (82300594) and the Institutional Foundation of The First Affiliated Hospital of Xi'an Jiaotong University (2022QN‐07).

## CONFLICT OF INTEREST STATEMENT

The authors declare no competing interests.

## Supporting information


**DATA S1:** Supporting Information.

## Data Availability

The data that support the findings of this study are available from the corresponding author upon reasonable request.
