# Supplementary material for: 4‐Octyl itaconate blocks GSDMB‐mediated pyroptosis and restricts inflammation by inactivating granzyme A
Source: Cell Prolif. 2024 Jul 9;57(12):e13711. doi: 10.1111/cpr.13711 (PMC11628737; doi:10.1111/cpr.13711)
Supplement: Supplementary file 1 — DATA S1: Supporting Information. [file CPR-57-e13711-s001.pdf]

Supplementary Information

4-Octyl itaconate blocks GSDMB-mediated pyroptosis and restricts inflammation by inactivating granzyme A

Wenbin Gong, Hangyu Fu, Kui Yang, Tao Zheng, Kun Guo, Wei Zhao

Correspondence to: Tao Zheng, email: jefferyzheng@yeah.net; Kun Guo, email: guokunmedical@126.com; Wei Zhao, email: zhaowei9803@xjtufh.edu.cn.

This PDF file includes:

Supplementary Figures 1 to 6.

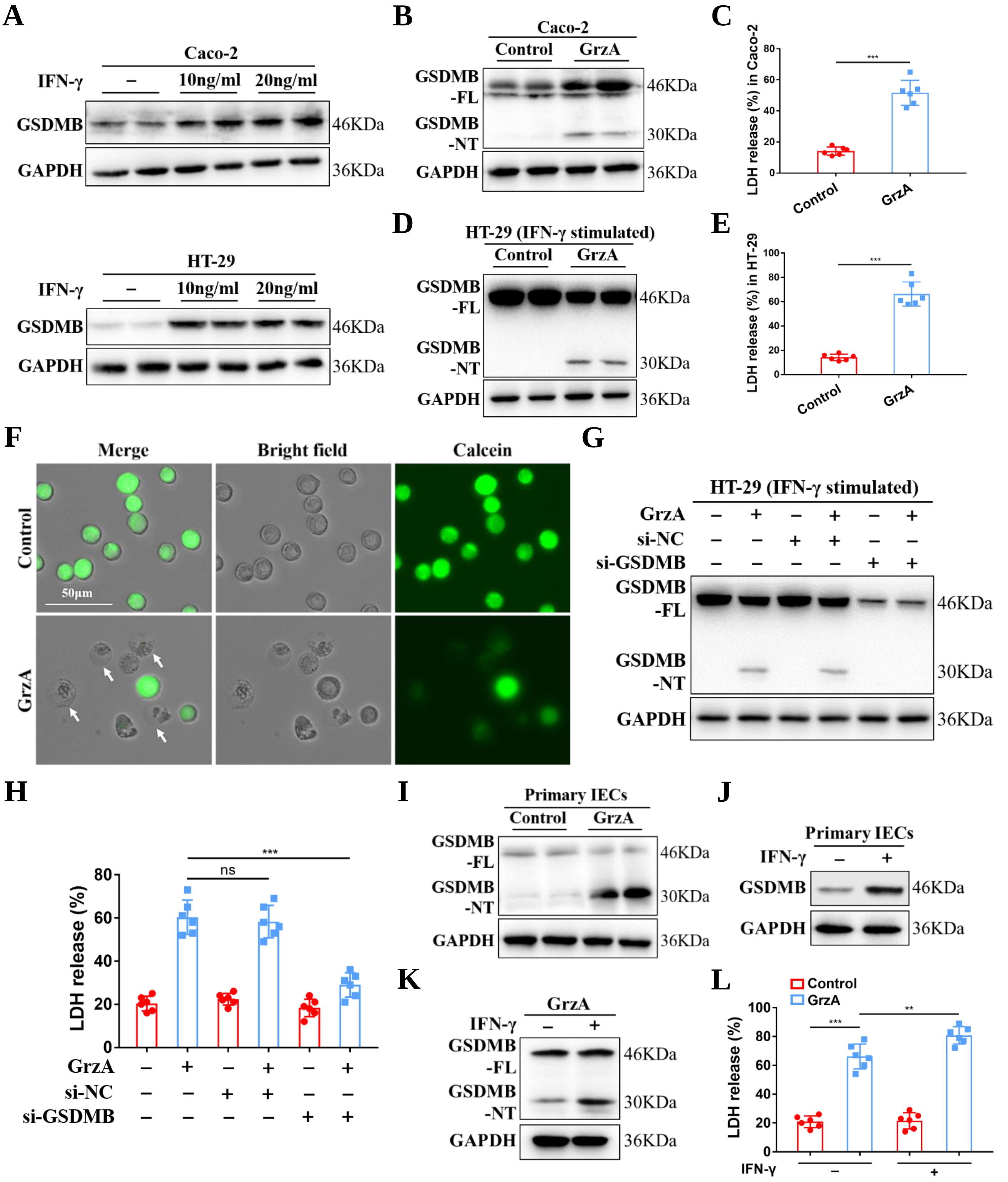

A
Caco-2
IFN-γ – 10ng/ml 20ng/ml
GSDMB 46KDa
GAPDH 36KDa
HT-29
IFN-γ – 10ng/ml 20ng/ml
GSDMB 46KDa
GAPDH 36KDa
B
Caco-2
Control GrzA
GSDMB -FL 46KDa
GSDMB -NT 30KDa
GAPDH 36KDa
C
LDH release (%) in Caco-2
Control GrzA
***
D
HT-29 (IFN-γ stimulated)
Control GrzA
GSDMB -FL 46KDa
GSDMB -NT 30KDa
GAPDH 36KDa
E
LDH release (%) in HT-29
Control GrzA
***
F
Merge Bright field Calcein
Control
GrzA
50μm
G
HT-29 (IFN-γ stimulated)
GrzA – + – + – +
si-NC – – + + – –
si-GSDMB – – – – + +
GSDMB -FL 46KDa
GSDMB -NT 30KDa
GAPDH 36KDa
H
LDH release (%)
***
ns
GrzA – + – + – +
si-NC – – + + – –
si-GSDMB – – – – + +
I
Primary IECs
Control GrzA
GSDMB -FL 46KDa
GSDMB -NT 30KDa
GAPDH 36KDa
J
Primary IECs
IFN-γ – +
GSDMB 46KDa
GAPDH 36KDa
K
GrzA
IFN-γ – +
GSDMB -FL 46KDa
GSDMB -NT 30KDa
GAPDH 36KDa
L
Control
GrzA
LDH release (%)
***
**
IFN-γ – +

Supplementary figure 1. GSDMB triggers pyroptosis of IECs and IFN-γ primes GSDMB expression to promote such cell death. (A) Western blot analysis of GSDMB in Caco-2 and HT-29 cells after stimulated with IFN-γ (10 mg/ml and 20 mg/ml). (B, D) Cleavage of GSDMB in response to electroporation of purified GrzA in Caco-2 and HT-29 cells. (C, E) LDH release activity from the media of these two cell lines after GrzA electroporation. (F) Representative images of IFN-γ-stimulated HT-29 cells after a 6h of electroporation with GrzA. HT-29 cells were preloaded with calcein AM dye. Arrows mark pyroptotic cells. Scale bar, 50 μm. (G, H) Analysis of GSDMB cleavage and LDH release in HT-29 cells electroporated with GrzA after knockdown of GSDMB with siRNA. (I) Analysis of GSDMB in response to electroporation of purified GrzA in primary IECs. (J, K) IFN-γ promoted GSDMB expression and its proteolytic cleavage in primary IECs when electroporating with GrzA. (L) LDH activity in IFN-γ-stimulated primary IECs after 6h of electroporation with GrzA. Data were displayed as mean values ± SD. $^{**}p < 0.01$, $^{***}p < 0.001$; ns, no significance.

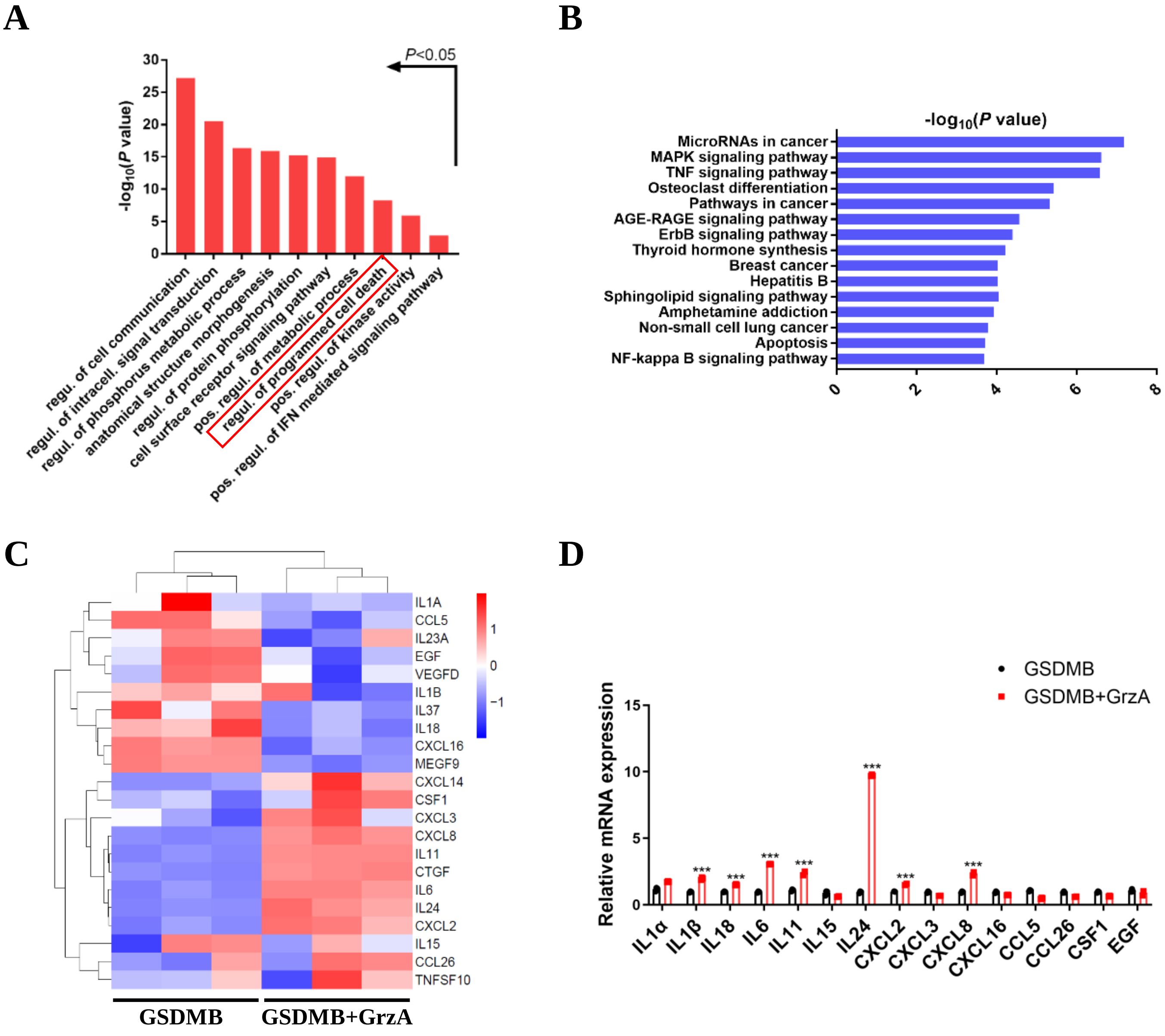

A
P<0.05
-log10(P value)
30
25
20
15
10
5
0
regu. of cell communication
regul. of intracell. signal transduction
regul. of phosphorus metabolic process
anatomical structure morphogenesis
regul. of protein phosphorylation
cell surface receptor signaling pathway
pos. regul. of metabolic process
regul. of programmed cell death
pos. regul. of kinase activity
pos. regul. of IFN mediated signaling pathway
B
-log10(P value)
MicroRNAs in cancer
MAPK signaling pathway
TNF signaling pathway
Osteoclast differentiation
Pathways in cancer
AGE-RAGE signaling pathway
ErbB signaling pathway
Thyroid hormone synthesis
Breast cancer
Hepatitis B
Sphingolipid signaling pathway
Amphetamine addiction
Non-small cell lung cancer
Apoptosis
NF-kappa B signaling pathway
0
2
4
6
8
C
IL1A
CCL5
IL23A
EGF
VEGFD
IL1B
IL37
IL18
CXCL16
MEGF9
CXCL14
CSF1
CXCL3
CXCL8
IL11
CTGF
IL6
IL24
CXCL2
IL15
CCL26
TNFSF10
1
0
−1
GSDMB
GSDMB+GrzA
D
GSDMB
GSDMB+GrzA
Relative mRNA expression
15
10
5
0
***
IL1α
IL1β
IL18
IL6
IL11
IL15
IL24
CXCL2
CXCL3
CXCL8
CXCL16
CCL5
CCL26
CSF1
EGF

Supplementary figure 2. RNA-seq reveals the inflammatory characteristics of IECs when cleaved by GrzA. (A) Gene ontology (GO) analysis indicated biological processes that were significantly upregulated in response to GrzA electroporation compared with no GrzA treatment in GSDMB-expressing IECs. (B) Kyoto Encyclopedia of Genes and Genomes (KEGG) pathway analysis showed cleavage of GSDMB led to activation of some inflammatory pathways in IECs, particularly TNF signaling pathway. (C) Heat map presented the expression of genes involved in cytokines and chemokines in GSDMB-expressing IECs with GrzA electroporation compared to that without GrzA intervention. (D) qRT-PCR analysis of cytokines and chemokines mRNA in GrzA-treated or untreated GSDMB-expressing IECs. Data were displayed as mean values ± SD. ***$p < 0.001$.

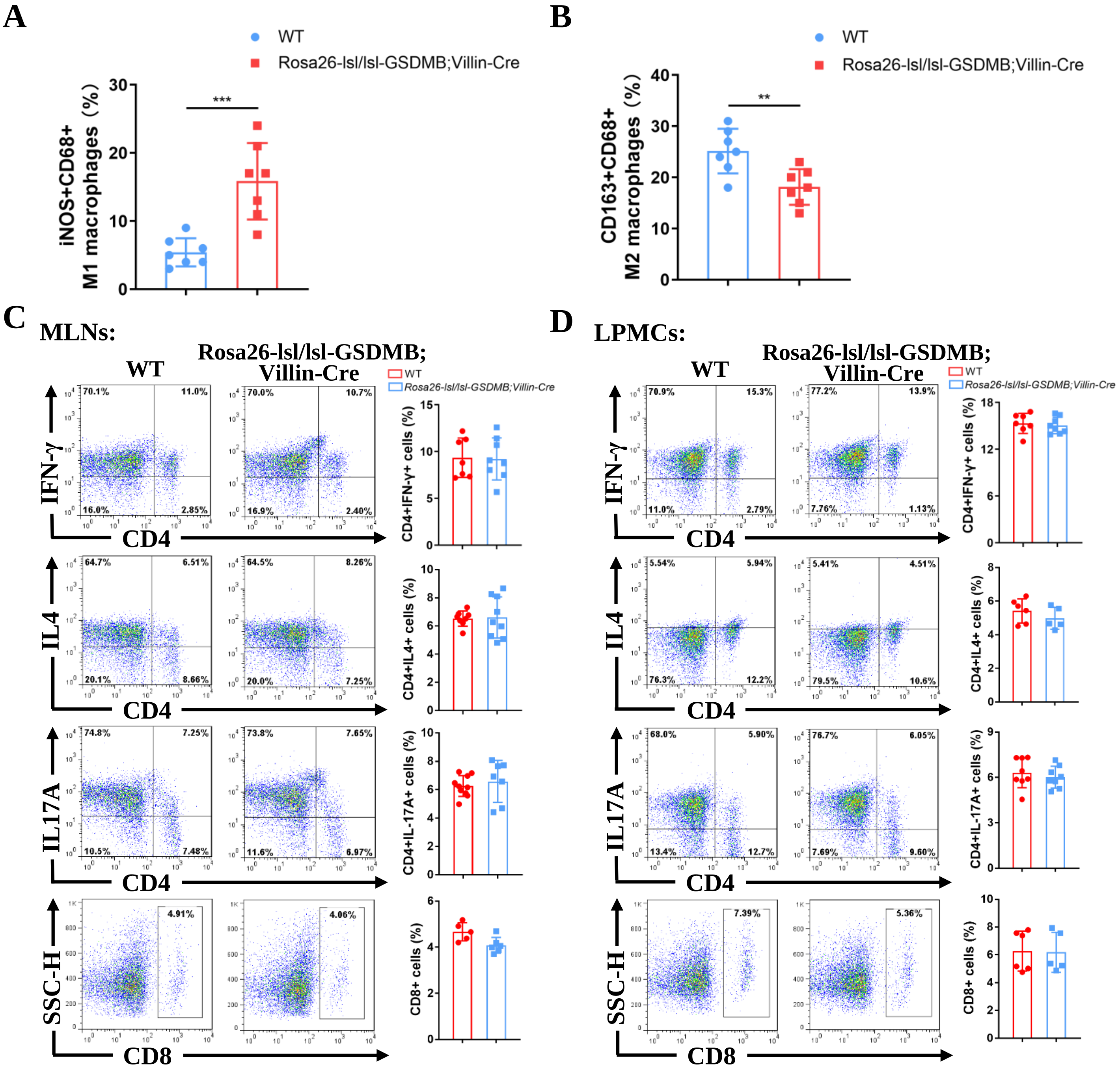
A
WT
Rosa26-lsl/lsl-GSDMB;Villin-Cre
iNOS+CD68+
M1 macrophages（%）
***
B
WT
Rosa26-lsl/lsl-GSDMB;Villin-Cre
CD163+CD68+
M2 macrophages（%）
**
C
MLNs:
WT
Rosa26-lsl/lsl-GSDMB;
Villin-Cre
IFN-γ
IL4
IL17A
SSC-H
CD4
CD8
CD4+IFN-γ+ cells (%)
CD4+IL4+ cells (%)
CD4+IL-17A+ cells (%)
CD8+ cells (%)
D
LPMCs:
WT
Rosa26-lsl/lsl-GSDMB;
Villin-Cre
IFN-γ
IL4
IL17A
SSC-H
CD4
CD8
CD4+IFN-γ+ cells (%)
CD4+IL4+ cells (%)
CD4+IL-17A+ cells (%)
CD8+ cells (%)

Supplementary figure 3. Transgenic expression of epithelial GSDMB leads to increased susceptibility to acute colitis. (A, B) The proportion of M1 and M2 macrophages in the colonic tissues of Rosa26-lsl/lsl-GSDMB;Villin-Cre and WT mice analyzed by immunofluorescence staining. (C, D) Flow cytometry analysis of IFN-γ+ (Th1), IL-4+ (Th2) IL-17A+ (Th17) CD4+ T cells and CD8+ T cells in the MLNs and LPMCs of Rosa26-lsl/lsl-GSDMB;Villin-Cre and WT mice after DSS insult. Values were expressed as mean± SD at least three independent experiments. **$p < 0.01$, ***$p < 0.001$.

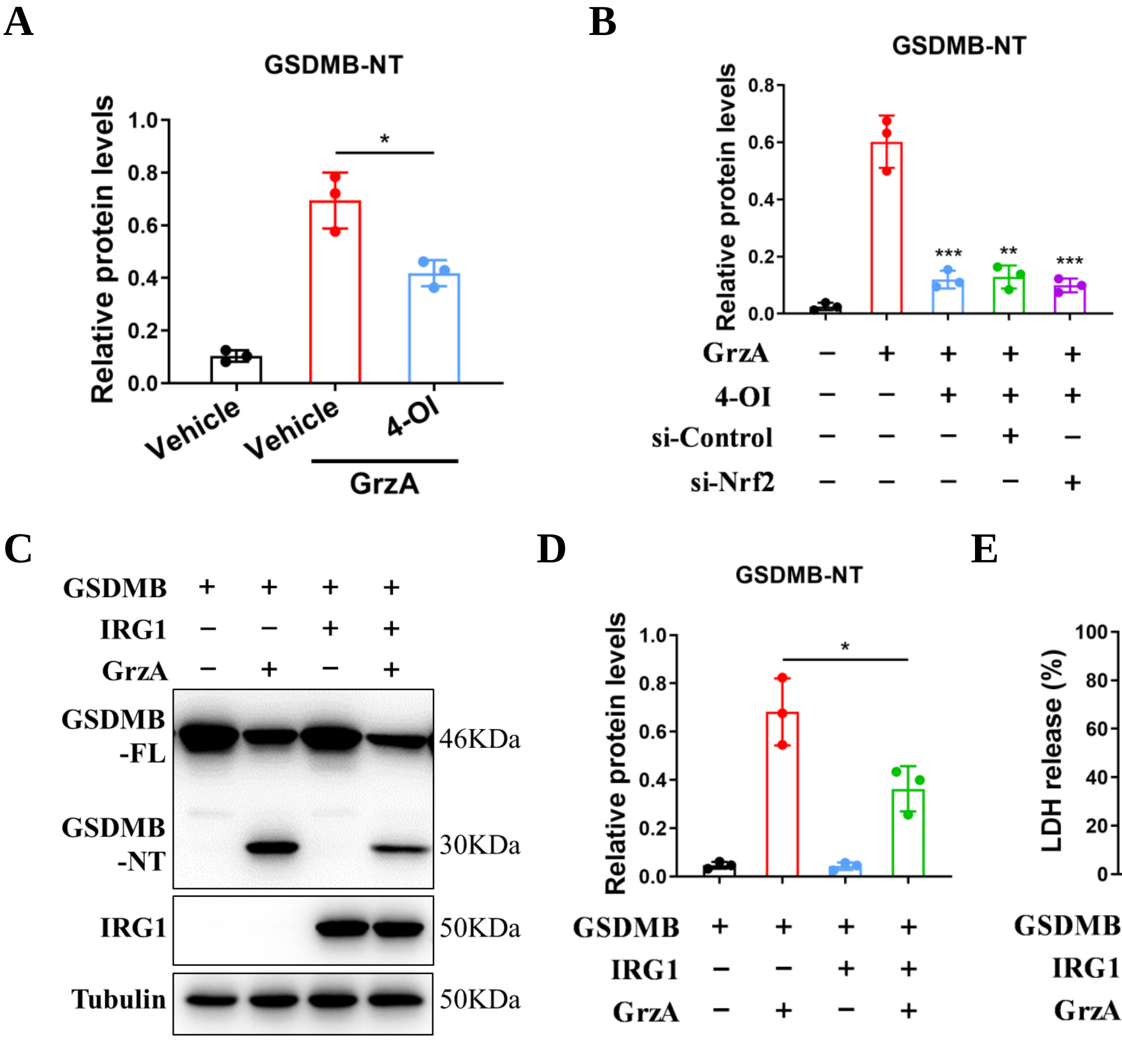
A
GSDMB-NT
Relative protein levels
1.0
0.8
0.6
0.4
0.2
0.0
*
Vehicle
Vehicle
4-OI
GrzA
B
GSDMB-NT
Relative protein levels
0.8
0.6
0.4
0.2
0.0
***
**
***
GrzA − + + + +
4-OI − − + + +
si-Control − − − + −
si-Nrf2 − − − − +
C
GSDMB + + + +
IRG1 − − + +
GrzA − + − +
GSDMB
-FL
46KDa
GSDMB
-NT
30KDa
IRG1
50KDa
Tubulin
50KDa
D
GSDMB-NT
Relative protein levels
1.0
0.8
0.6
0.4
0.2
0.0
*
GSDMB + + + +
IRG1 − − + +
GrzA − + − +
E
LDH release (%)
100
80
60
40
20
0
**
GSDMB + + + +
IRG1 − − + +
GrzA − + − +

Supplementary figure 4. Endogenous itaconate inhibits GSDMB-rendered pyroptosis. (A) Relative protein levels of GSDMB-NT in human primary IECs after 4-OI treatment. (B) Relative protein levels of GSDMB-NT after knockdown of Nrf2. (C, D) Western blot analysis of GSDMB cleavage in GSDMB-expressing 293T cells and (E) LDH release activity from the media when overexpressing IRG1. Data were displayed as mean values ± SD. *p <0.05, **p <0.01, ***p <0.001.

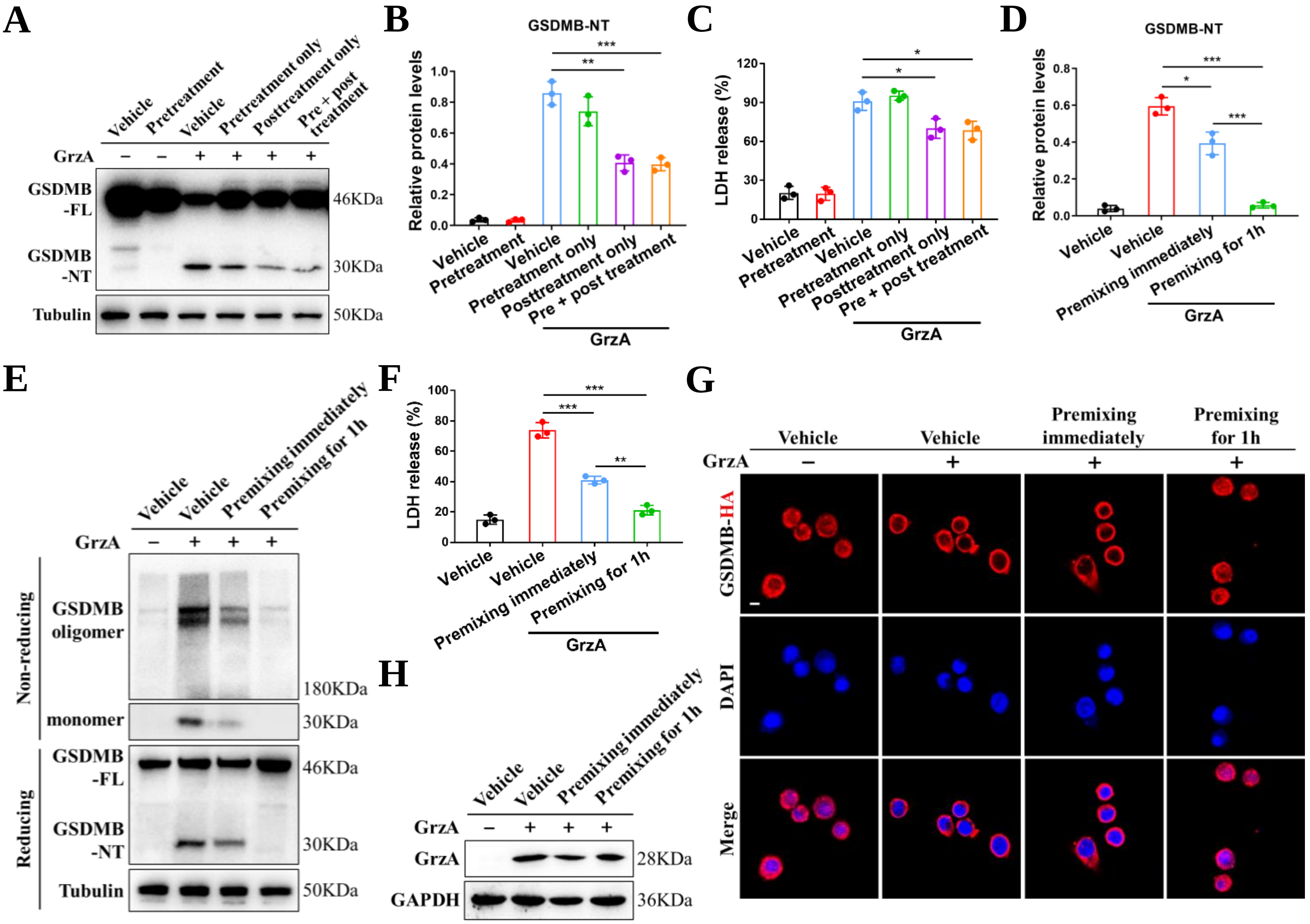

A
Vehicle
Pretreatment
Vehicle
Pretreatment only
Posttreatment only
Pre + post treatment
GrzA
GSDMB -FL
GSDMB -NT
Tubulin
46KDa
30KDa
50KDa
B
GSDMB-NT
Relative protein levels
C
LDH release (%)
D
Premixing immediately
Premixing for 1h
E
Non-reducing
Reducing
GSDMB oligomer
monomer
180KDa
F
G
GSDMB-HA
DAPI
Merge
Premixing immediately
Premixing for 1h
H
GAPDH
28KDa
36KDa

Supplementary figure 5. 4-OI inhibits pyroptosis not through interaction with GSDMB. (A, B) Cleavage of GSDMB and (C) LDH release activity in GSDMB-expressing 293T cells when exposed to 4-OI in different phases. (D, E) GSDMB oligomerization and monomer and (F) LDH release activity in GSDMB-expressing 293T cells when premixed with 4-OI immediately or preincubated for 1 hour before electroporation with GrzA. (G) The HA-tagged GSDMB was overexpressed in 293T cells and the membranous localization of GSDMB was observed. Scale bar, 10 μm. (H) Western blot analysis of GrzA protein in GSDMB-expressing 293T cells when co-incubated with 4-OI. Values were expressed as mean ± SD at least three independent experiments. *p <0.05, **p <0.01, ***p <0.001.

A

B

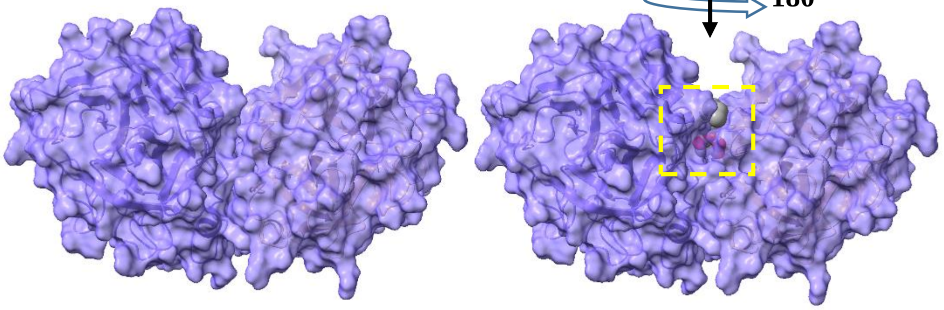

C

Mode	Affinity (kcal/mol)	Dist from rmsd l. b.	Best mode rmsd u. b.
1	-5.2	0.000	0.000
2	-4.6	5.622	7.889
3	-4.6	3.813	8.833

D

CurPocket ID	Vina score	Cavity volume (Å^3)	Center (x, y, z)	Docking size (x, y, z)
C1	-5.5	992	-12, -6, -10	22, 22, 22
C5	-5.3	91	-5, 2, -18	22, 22, 22
C4	-3.9	101	-8, 14, 8	22, 22, 22
C2	-3.8	203	-17, 11, 12	22, 22, 22
C3	-3.8	106	-23, 0, 1	22, 22, 22

E

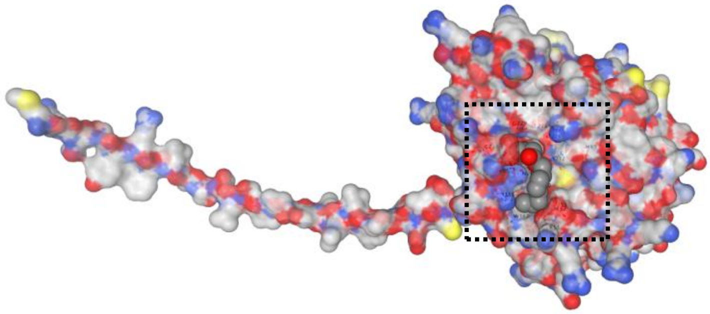

F

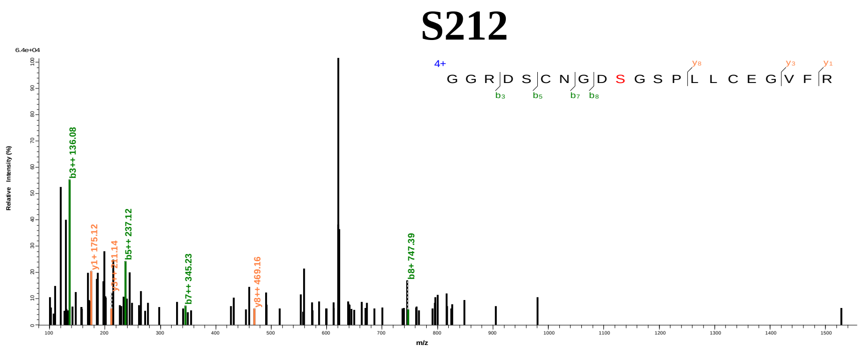

Supplementary figure 6. 4-OI directly modifies Granzyme A to block GSDMB-rendered pyroptosis. (A) Structure of GrzA dimer. (B, C) The possible interaction between GrzA dimer and 4-OI. (D, E) The predicted interaction between 4-OI and GrzA via protein-ligand docking tool CB-Dock2. (F) Tandem mass spectrometry spectrum of GrzA peptide following 4-OI treatment (0.5 mM, 6 h).
